# Rapid Formation of Microbe-Oil Aggregates and Changes in Community Composition in Coastal Surface Water Following Exposure to Oil and the Dispersant Corexit

**DOI:** 10.3389/fmicb.2018.00689

**Published:** 2018-04-11

**Authors:** Shawn M. Doyle, Emily A. Whitaker, Veronica De Pascuale, Terry L. Wade, Anthony H. Knap, Peter H. Santschi, Antonietta Quigg, Jason B. Sylvan

**Affiliations:** ^1^Department of Oceanography, Texas A&M University, College Station, TX, United States; ^2^Geochemical and Environmental Research Group, Texas A&M University, College Station, TX, United States; ^3^Department of Marine Science, Texas A&M University at Galveston, Galveston, TX, United States; ^4^Department of Marine Biology, Texas A&M University at Galveston, Galveston, TX, United States

**Keywords:** deepwater horizon, oil and corexit®, micro-aggregate, marine oil-snow, MOSSFA

## Abstract

During the Deepwater Horizon (DWH) oil spill, massive quantities of oil were deposited on the seafloor via a large-scale marine oil-snow sedimentation and flocculent accumulation (MOSSFA) event. The role of chemical dispersants (e.g., Corexit) applied during the DWH oil spill clean-up in helping or hindering the formation of this MOSSFA event are not well-understood. Here, we present the first experiment related to the DWH oil spill to specifically investigate the relationship between microbial community structure, oil and Corexit®, and marine oil-snow in coastal surface waters. We observed the formation of micron-scale aggregates of microbial cells around droplets of oil and dispersant and found that their rate of formation was directly related to the concentration of oil within the water column. These micro-aggregates are potentially important precursors to the formation of larger marine oil-snow particles. Therefore, our observation that Corexit® significantly enhanced their formation suggests dispersant application may play a role in the development of MOSSFA events. We also observed that microbial communities in marine surface waters respond to oil and oil plus Corexit® differently and much more rapidly than previously measured, with major shifts in community composition occurring within only a few hours of experiment initiation. In the oil-amended treatments without Corexit®, this manifested as an increase in community diversity due to the outgrowth of several putative aliphatic- and aromatic-hydrocarbon degrading genera, including phytoplankton-associated taxa. In contrast, microbial community diversity was reduced in mesocosms containing chemically dispersed oil. Importantly, different consortia of hydrocarbon degrading bacteria responded to oil and chemically dispersed oil, indicating that functional redundancy in the pre-spill community likely results in hydrocarbon consumption in both undispersed and dispersed oils, but by different bacterial taxa. Taken together, these data improve our understanding of how dispersants influence the degradation and transport of oil in marine surface waters following an oil spill and provide valuable insight into the early response of complex microbial communities to oil exposure.

## Introduction

On April 20th, 2010 the DWH drilling rig exploded in the northern Gulf of Mexico, resulting in the largest oil spill accident in the history of the U.S. petroleum industry (Atlas and Hazen, 2011). Over the following 87 days, an estimated 650,000–780,000 m^3^ (4.1–4.9 million barrels) of crude oil was released (McNutt et al., 2012; Reddy et al., 2012) with an estimated 60% reaching the sea surface. During the spill, a large formation of mucus-rich, oil-associated marine snow was observed around the accident site which subsequently aggregated and sank to the seafloor over a period of several weeks (Passow et al., 2012). As they sank, marine snow aggregates captured oil droplets from the sea surface and upper water column and transported large quantities of oil to the seafloor. This phenomenon, described as a marine oil snow sedimentation and flocculent accumulation (MOSSFA) event, was the result of microorganisms releasing large amounts of exopolymeric substances (EPS) in response to the oil. Similar events were observed at the IXTOC I blowout in the southern Gulf of Mexico in 1979 (Patton et al., 1981), and possibly also at the Santa Barbara blowout in 1969 (Vonk et al., 2015).

EPS are high molecular-weight polymers composed primarily of polysaccharides and proteins which are excreted by a variety of bacteria and phytoplankton species (see recent review, Quigg et al., 2016). EPS, especially in marine environments, often contain charged (e.g., uronic acid) or amphiphilic moieties which enable them to interface with and/or emulsify hydrophobic organic chemicals such as hydrocarbons. Transparent exopolymer particles (TEP), an operationally defined subgroup of EPS, were observed in surface water aggregates as well as in sediment traps associated with marine oil snow after the DWH spill (Passow et al., 2012). These materials increase the bioavailability of otherwise highly insoluble hydrocarbon substrates to biodegradation and as such play a critical role in the degradation of oil in marine surface waters and its transport to the seafloor.

Chemical dispersants (e.g., Corexit 9527; Corexit 9500) have historically been applied to marine oil spills in an effort to move more oil from the sea surface into the water column, decreasing the amount of oil that washes up on shore by breaking oil down into smaller droplets (Lessard and DeMarco, 2000). This also increases the total amount of oil available to hydrocarbon-degrading microorganisms and provides increased surface area on the smaller oil droplets for microorganisms to utilize. However, the usage of dispersants as a remediation method remains controversial as the impact of their application on microbial communities remains poorly resolved, especially in surface waters. For example, some recent studies have demonstrated that some hydrocarbon degrading bacteria are inhibited by chemical dispersants (Hamdan and Fulmer, 2011; Kleindienst et al., 2015a,b) and thus their usage ultimately delays microbial crude oil biodegradation. On the other hand, numerous studies of various marine oil spills over the last 30 years have demonstrated dispersant usage lowers overall environmental impact, especially in coastal and inter-tidal environments where the risk of oil coming ashore is highest (Ballou et al., 1989; Lewis and Aurand, 1997; Lessard and DeMarco, 2000). Some experiments have revealed increased small-scale aggregation of prokaryotes in the presence of Corexit (Baelum et al., 2012; Kleindienst et al., 2015b; Quigg et al., 2016), though it is unknown if those micro-aggregates have a different community composition or hydrocarbon oxidation potential than non-dispersant treated communities. The abundance and nature of these micro-aggregates *in situ* is also unknown.

In this study, we prepared mesocosms with coastal water to investigate the responses of natural microbial communities to the water accommodated fraction (WAF) of oil with and without the dispersant Corexit. Mesocosms are ideal as they have the great advantage that they allow detailed investigations into biological, chemical, and physical processes and parameters, something that cannot be accomplished in field research where relationships between processes usually only allow for correlations between parameters. We present here a description of the abundance, diversity, and enzymatic activity (β-galactosidase) of the mesocosm prokaryotic communities based on cell counts of single microbes and micro-aggregates, analysis of amplified 16S rRNA genes, and quantification of the cytochrome P450 alkane hydroxylase gene. Previous studies have focused primarily on the response of the deep ocean microbial communities affected by the DWH plume, which formed at ~1100–1300 m water depth (Hazen et al., 2010; Baelum et al., 2012; Redmond and Valentine, 2012; Rivers et al., 2013; Crespo-Medina et al., 2014; Kleindienst et al., 2016a). We here focus on surface waters, where interactions between light, warmer temperatures, phytoplankton, and prokaryotic microbes all combine to drive hydrocarbon biodegradation.

## Materials and methods

### Mesocosm setup and sampling

Seawater (salinity = 31‰) was collected on October 17, 2015 from a pipeline located ~100 m offshore south of Galveston, TX, in the Gulf of Mexico at 29.2726°N, 94.8126°W and transferred to a holding tank in the Texas A&M University at Galveston Sea Life Facility (TAMUG SLF), covered, and stored at room temperature overnight. Four treatments (Figure S1) were prepared in triplicate tanks. Control tanks were filled with untreated seawater only. WAF of oil was prepared by adding 25 mL (5 mL ~ every 30 min for 2.5 h) of Macondo surrogate oil into 130 L of seawater in a baffled recirculation tank (BRT) (Knap et al., 1983; Wade et al., 2017) and allowed to mix for ~24 h. This was performed in duplicate BRTs to make sufficient WAF for three mesocosm tanks. The WAF was then drawn from the bottom of the BRT and introduced into the WAF mesocosm tanks (87 L each) and mixed. Non-accommodated oil remained as a surface slick in the BRTs. In order to make a chemically enhanced water accommodated fraction (CEWAF) of oil, Corexit was mixed with oil in a ratio of 1:20 and 25 mL of this mixture (5 mL every 30 min for 2.5 h) added to 130 L of seawater which was mixed for ~24 h prior to being transferred to the mesocosm tanks. Diluted CEWAF (DCEWAF) was prepared by mixing 9 L of CEWAF with 78 L of the original seawater for a total volume of 87 L. CEWAF was prepared in triplicate BRTs to ensure sufficient volume for all CEWAF and DCEWAF mesocosm tanks.

Plankton were collected from the TAMUG dock using a net (≥63 μm), transferred into polycarbonate bottles, and then added (2 L) into the tanks and stirred immediately prior to starting the experiments. Banks of full-spectrum (UV-Vis 375–750 nm, <50 μmol photons m^−2^ s^−1^) fluorescent lamps (Sylvania GRO-LUX®) were placed directly beside (~10–15 cm) each of the glass mesocosm tanks and a 12:12 h light/dark cycle employed (Figure S1). Mesocosm tanks were open to the atmosphere and no stirring was used after experiment initiation. Further details on the preparation of WAF, CEWAF, and DCEWAF are available in Wade et al. (2017).

Starting at time-point zero and every 12 h thereafter, 1 L of water was collected from each mesocosm in clean, opaque Nalgene bottles with PTFE-lined diaphragm pumps. Cell count samples (10 mL) were fixed with formalin (final concentration 2%) and stored at 4°C until analysis. For molecular biology analyses, 150 mL were pre-filtered through 10 μm filters to remove most eukaryotic cells followed by filtration onto 47 mm 0.22 μm Supor PES filter membranes (Pall; Port Washington, USA). All filters were stored at −80°C until DNA extraction.

### Estimated oil equivalence (EOE)

During the time-course of the experiment, estimated oil equivalents (EOE) were determined by fluorescence (Wade et al., 2011) using Macondo surrogate oil as a standard to produce calibration curves at 5–7 concentrations. Water samples (5–20 mL) were extracted with 5 mL of dichloromethane. An aliquot of the extract was placed in a cuvette for fluorescence analyses (Horiba Scientific Aqualog Fluorometer). The EOE were determined from the calibration curve (Wade et al., 2011). Samples with fluorescence responses that exceeded the calibration curve were diluted so that their fluorescence was within the calibration range.

### Total cell abundance and micro-aggregate counts

Direct cell counts were performed with an epifluorescence microscope (Zeiss Axio Imager.M2) after staining the fixed samples with DAPI (45 μM final concentration) for 5 min in the dark and filtering them onto 25 mm, 0.2 μm black polycarbonate filters. Each filter was mounted on a glass microscope slide with coverslip using two drops of anti-fade solution (90 mM *p*-phenylenediamine and 45% glycerol dissolved in phosphate buffered saline, filter sterilized). A 100 × 100 μm ocular counting grid was used at 1,000 × magnification for cell counts. Due to their much larger size, micro-aggregates were quantified at 400 × magnification. For total cell abundance, if part of a micro-aggregate was within the field of view, all visible cells were counted. For micro-aggregate abundance, the presence of an aggregate was counted, not the number of cells present per aggregate. Aggregates were defined as groups of cells in clumps 10–200 μm in diameter, often found gathered around drops of oil (Figure S2). Nested ANOVAs were conducted to test for significant differences in cell abundances over time and between treatments.

### β-galactosidase assays

Beta-galactosidase measurements were performed on whole water and <10 μm size fractions by collecting 10 mL sample and adding 4-methylumbelliferyl β-D-galactopyranoside (Sigma-Aldrich; St.Louis, USA) at a final concentration of 150 μM. Immediately after substrate addition and at two subsequent time points, typically an hour and 2 h, 1 mL sample was removed and frozen for later analysis. Upon thawing, fluorescence was quantified using a Tecan Spark 10 M multimode microplate reader with excitation and emission wavelengths set at 380/20 and 454/20 nm, respectively. Amount of substrate cleaved was calculated by fitting raw fluorescence to a standard curve of 4-methylumbellierone (Sigma-Aldrich) included in every plate read. To obtain optimal sample pH for fluorescence quantification, 50 mM sodium borate was added to both samples and standards prior to reading.

### DNA extraction, 16S rRNA sequencing, and qPCR

Total DNA was extracted from filters using FastDNA Spin kits (MP Biomedical; Santa Ana, California) and stored at −20°C until PCR amplification. Three sample-free filters were processed as protocol blanks in order to control for the possible presence of DNA contamination in the extraction kits and PCR reagents (Salter et al., 2014). The hyper-variable V4 region of the 16S rRNA gene was PCR amplified from the DNA extracts with GoTaq Flexi DNA Polymerase (Promega, Fitchburg, USA) using a methodology similar to that described by Caporaso et al. (2012). Each sample was amplified in triplicate 25 μL reactions with the following cycling parameters: 95°C for 3 min, 30 cycles of 95°C for 45 s, 50°C for 60 s, and 72°C for 90 s, and a final elongation step at 72°C for 10 min. All amplifications were performed using the 515F-806R primer pair (10 μM each) modified to include recently published revisions that reduce bias against the *Crenarchaeota* and *Thaumarchaeota* lineages as well as the SAR11 bacterial clade (Apprill et al., 2015; Parada et al., 2016). The primer pair was additionally modified to include Golay barcodes and adapters for Illumina MiSeq sequencing. Final primer sequences are detailed in Walters et al. (2016). Following amplification, the triplicate products were combined together and run on a 1.5% agarose gel to assess amplification success and relative band intensity. Amplicons were then quantified with the QuantiFluor dsDNA System (Promega), pooled at equimolar concentrations, and purified with an UltraClean PCR Clean-Up Kit (MoBio Laboratories; Carlsbad, USA). The purified library, along with aliquots of the three sequencing primers, were sent to the Georgia Genomics Facility (Athens, GA, USA) for MiSeq sequencing (v2 chemistry, 2 × 250 bp).

Primers *P450fw1* (GTSGGCGGCAACGACACSAC) and *P450rv3* (GCASCGGTGGATGCCGAAGCCRAA) were used to obtain PCR products for cytochrome P450 alkane hydroxylase (van Beilen et al., 2006). PCR products were ligated into competent *E. coli* cells and cloned using the TOPO TA Cloning Kit (Thermo Fisher Scientific; Waltham, USA). Plasmid extractions were performed using Qiagen Mini-prep Kits. Sequencing of seven random clones verified the success of the cloning reaction and exact size and sequence of the targeted PCR products were confirmed with BlastX. A mixture of five P450 clone sequences were used to create the standard, three Alphaproteobacteria and one Gammaproteobacteria. qPCR was carried out for all samples in triplicate 25 μL reactions using 12.5 μL of iQ SYBR Green Supermix (Bio-Rad; Hercules, USA) and 10 μM of each primer with the following cycling parameters: 95°C for 3 min, 40 cycles of 95°C for 10 s, 57°C for 30 s, and 72°C for 45 s, and a final elongation step at 72°C for 45 s. A melt-curve was included at the end of each PCR cycle to confirm only a single product was amplified. Sequences of these standards are available on GenBank under accession numbers MF962916-MF962919.

### Molecular biology analysis

Sequence read curation and processing were carried out using mothur v.1.36.1 (Schloss et al., 2009) following a modified version of the protocol described in Kozich et al. (2013). Paired reads were combined into contigs and quality filtered to exclude sequences containing ambiguous base calls or homopolymer runs longer than 8 bp. The merged contigs were then aligned with the SILVA non-redundant 16S rRNA reference dataset (v.123). To help mitigate the generation of spurious sequences, the aligned sequences were “pre-clustered,” allowing 1-bp difference per 100 bp of sequence (Huse et al., 2010). Chimeric sequences were identified with the UCHIME algorithm (Edgar et al., 2011) and removed from further processing and analysis. Sequences were then clustered into OTUs at 3% or less dissimilarity using the average neighbor method (Schloss and Westcott, 2011). As a final quality control measure, potential contaminant sequences were screened by removing any OTU from the dataset wherein 1% or greater of its sequences were sourced from any of the three protocol blank libraries.

We constructed 10,079,362 contigs with an average read length of 255 bp distributed across 87 barcoded samples from Illumina MiSeq paired-end 250-bp sequencing. After screening of sequences with ambiguous bases or long homopolymers, 694,269 unique sequences were used to generate an alignment. Further quality control measures (i.e., pre-clustering, alignment curation, and chimera removal) reduced the total library to 138,542 unique contigs which clustered into 9,920 OTUs. As a final curation step, OTUs in which 1% or greater of its member sequences were from a protocol blank library were considered likely background contamination and removed from the dataset. Twenty-six OTUs met this criteria for removal, resulting in a final curated dataset containing 9894 OTUs clustered from 6,230,128 sequences. Each library contained an average of 74,168 ± 18,927 sequences (Table S1). Rarefaction curves for all samples approached saturation (Figure S3), and together with high Good's Coverage values (>99%, Table S1) indicate that the remaining un-sampled diversity likely contained only rare taxa.

A consensus taxonomy for each OTU was assigned using a naïve Bayesian classifier (Wang et al., 2007) trained on the SILVA reference database (Quast et al., 2013) with a confidence threshold of 80%. The taxonomic assignments for OTUs of interest were then checked by classifying a representative sequence, selected as the most abundant within the OTU, with the Greengenes, RDP, SILVA, GenBank, and EZ-Taxon databases (Table S2). Diversity and richness indices (observed richness, Inverse Simpson, Chao1, ACE) were calculated from the average (1000 iterations) of rarefying libraries to the smallest library size (23,242 sequences). Patterns in microbial community structure were examined using non-metric multidimensional scaling (NMDS) based on the Bray-Curtis dissimilarity index (Bray and Curtis, 1957), and tested for significance using analysis of molecular variance, AMOVA (Excoffier et al., 1992).

### Network analysis

OTUs present at relative abundance ≥0.01% overall for at least 15 samples (out of the 28 averaged triplicate samples) and ≥0.05% for four samples from one treatment were included in a local similarity analysis (Ruan et al., 2006), generated in R using rcor.test. False positives were tested for using the R package q-value (Dabney et al., 2010) and only correlations where *p* < 0.005, *q* < 0.05, and the Pearson's correlation was >0.70 were subsequently used for network visualization in Cytoscape 3.5 (Shannon et al., 2003).

### Data

All MiSeq data presented in this study are publically available through the Gulf of Mexico Research Initiative Information and Data Cooperative (GRIIDC) at https://data.gulfresearchinitiative.org. DOIs: 10.7266/N72F7KGG (16S rRNA libraries, NCBI BioProject PRJNA320765), 10.7266/N7R78C8T (cell counts, micro-aggregate counts, and β-galactosidase activity), 10.7266/N7SB43TH (P450 qPCR dataset), 10.7266/N7HT2MDX (micro-aggregate micrographs).

## Results

### Cell and micro-aggregate abundance

**Figure 1:**
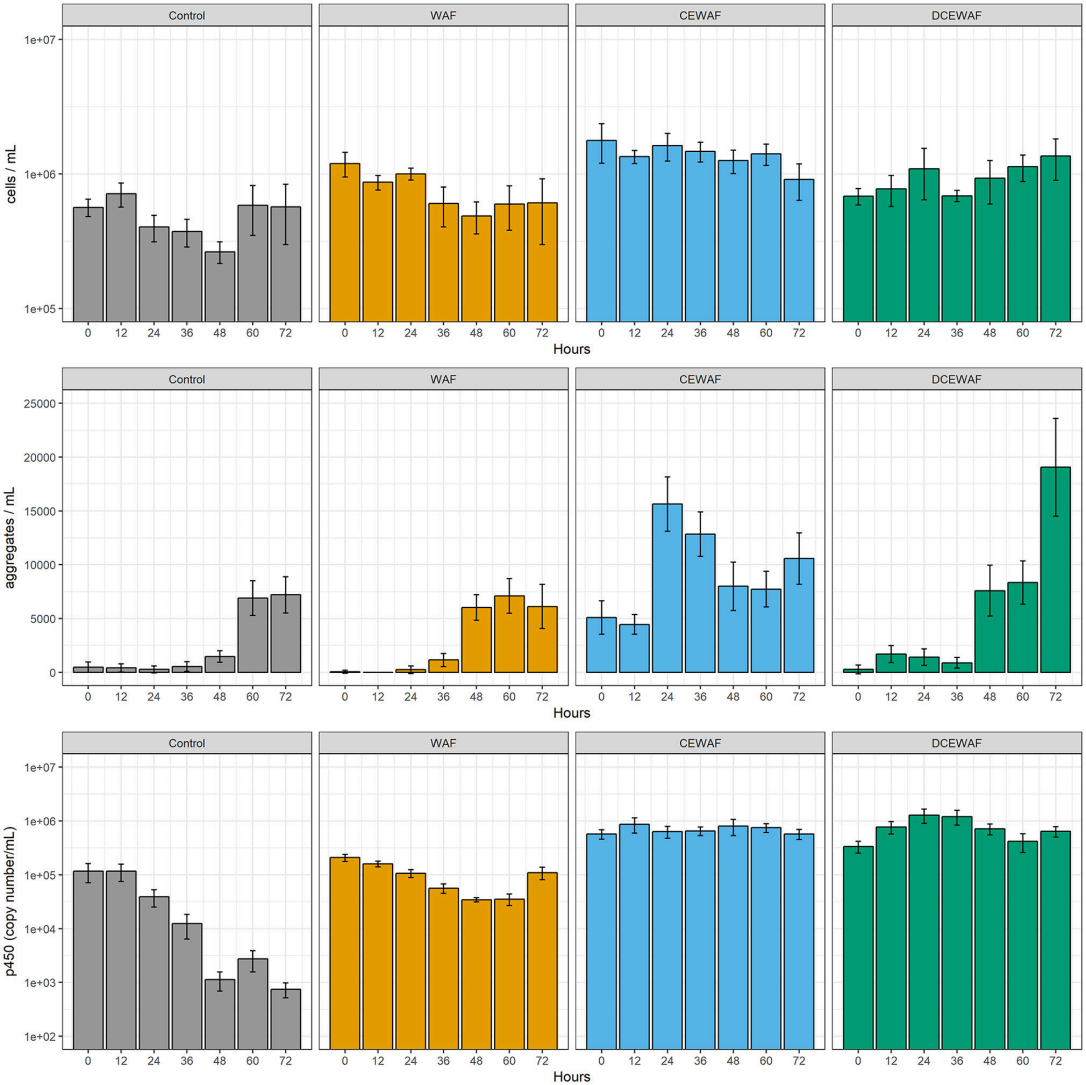
Cell abundance (top), micro-aggregate abundance (middle), and cytochrome P450 gene copy abundance (bottom) observed in the four mesocosm treatments. Columns represent the pooled mean of replicated measurements with error bars representing the pooled standard error.

Total cell abundances ranged from ~0.3 × 10^6^ mL^−1^ to ~1.8 × 10^6^ mL^−1^ (Figure 1, top) and did not vary significantly over time in any of the four experimental treatments (Control, WAF, CEWAF, and DCEWAF; see experimental Procedures for details). There were, however, statistically significant differences in cell abundances between the four treatments [*F*_(3, 24)_ = 17.246, *p* < 0.001]; but these differences, being at most less than an order of magnitude, were relatively small. For example, the CEWAF mesocosms had the highest overall cell abundances but averaged only ~3-fold more cells than those with the lowest cell abundance (Control). In contrast to cell abundances, the amount of micro-aggregates within the mesocosms increased several fold throughout the experimental time course (Figure 1, middle). Initially, micro-aggregates were nearly absent in all but the CEWAF mesocosms but then increased in abundance by between 15- and 139-fold after a lag period of ~36–48 h to maximum abundances of ~7,500 mL^−1^ in the Control and WAF treatments, ~16,000 mL^−1^ in the CEWAF treatment and ~19,000 mL^−1^ in the DCEWAF treatment. The CEWAF mesocosms appear to have already formed micro-aggregates before the first time point, likely during the experimental preparation and setup. Subsequently, the DCEWAF mesocosms were next to form micro-aggregates, followed shortly after by the WAF mesocosms. Micro-aggregates also formed in the Control mesocosms after appearing first in all other treatments.

### Cytochrome P450 alkane hydroxylase gene abundance

Quantification of the cytochrome P450 alkane hydroxylase gene is indicative of the microbial community's ability to degrade certain alkanes in each of the treatments. The P450 gene was present in all samples and time points (Figure 1, bottom). In the control treatment, P450 gene copy abundance was initially the lowest (1.16 ± 0.45 × 10^5^ copies mL^−1^; *M* ± *SE*) and further decreased ~150-fold over the course of the experiment. In contrast, the CEWAF and DCEWAF treatments had similar, elevated abundances of the P450 gene which did not decrease or vary significantly over time [*F*_(6, 35)_ = 0.47, *p* = 0.826]. *Post hoc* pairwise comparisons using Tukey's HSD test did not reveal any significant differences between the CEWAF and DCEWAF treatments at any time point. P450 gene abundances in the WAF treatments were intermediate to those in the Control and Corexit-amended treatments (i.e., CEWAF and DCEWAF). Similar to the Control treatments, P450 gene copy abundances in the WAF treatments also decreased over time, but to a much lesser extent.

### Estimated oil equivalence

**Figure 2:**
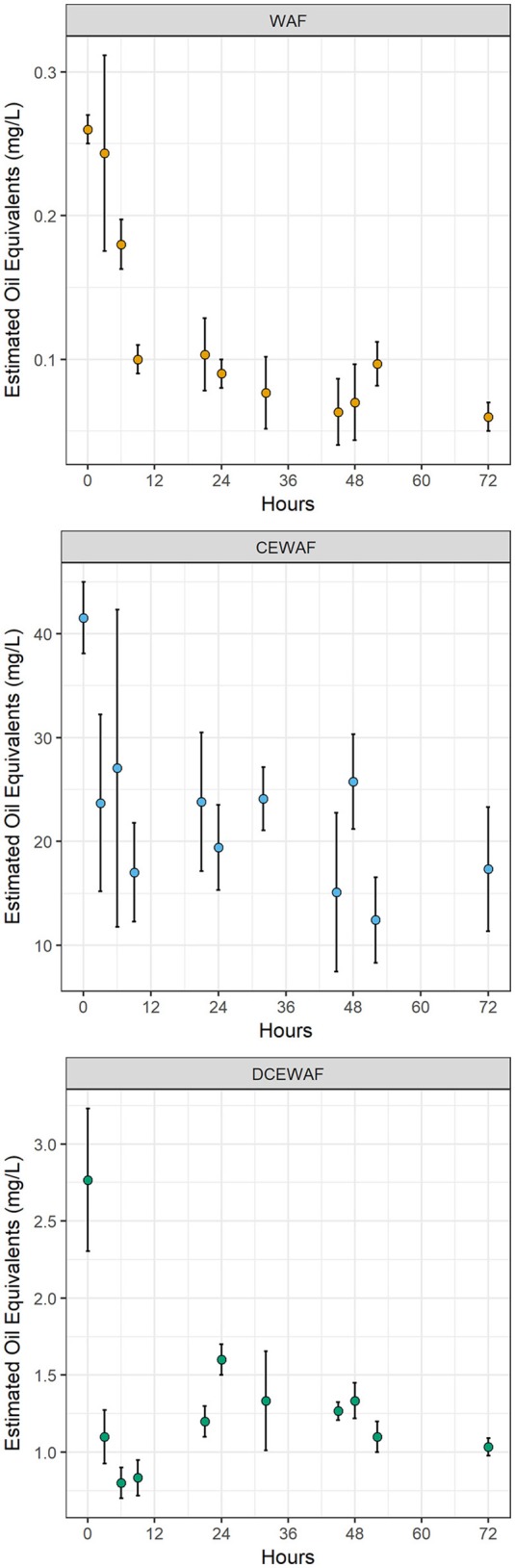
Estimated oil equivalents (EOE) within the WAF, CEWAF, and DCEWAF mesocosms. Points represent the average of triplicate measurements. Error bars represent standard deviation. EOE measurements in the Control mesocosms were below the detection limit and are not shown.

The concentration of water accommodated oil was lowest in the WAF treatments with an initial concentration of 0.26 ± 0.01 mg L^−1^ (*M* ± *SD*) which subsequently decreased to roughly 0.07 mg L^−1^ after 72 h (Figure 2). Within the CEWAF treatments, the use of dispersant allowed much more oil to be initially accommodated into the water column (41.5 ± 3.4 mg L^−1^). After 72 h, the EOE within these mesocosms decreased by roughly half, but still contained significantly more oil than either the WAF or DCEWAF treatments. The DCEWAF treatments contained an intermediate amount of oil between that of the WAF and CEWAF (initially 2.8 ± 0.5 mg L^−1^) and decreased to ~1.0 mg L^−1^ by the end of the experiment. EOE measurements in the Control treatments were below the detection limit (0.04 mg L^−1^).

### β-galactosidase activity

β-galactosidase activity did not differ significantly between the filtered and whole water fraction, indicating most of the activity was derived from the <10 μm size fraction (data not shown). Compared between treatments, β-galactosidase activity was similar in the CEWAF and DCEWAF mesocosms (710 ± 340 nM day^−1^; *M* ± *SD*) but decreased slowly over time in the Control and WAF treatments (Figure S4). β-galactosidase activity in the WAF mesocosms was substantially elevated for the first 60 h compared to the Control and other treatments, and then decreased at 72 h. At this final time point, β-galactosidase activity within the WAF mesocosms had decreased to such an extent that there was no longer a significant difference between any mesocosm treatment [*F*_(3, 6)_ = 3.486, *p* = 0.090].

### Microbial community composition and structure

Overall, the mesocosms' microbial communities exhibited relatively low diversity, with the top 30 most abundant operational taxonomic units (OTUs) containing ~86% of all sequences. *Alpha*- and *Gammaproteobacteria* were overwhelmingly the most abundant in all four mesocosm treatments, together comprising 89.1% (median, interquartile range = 80.4–94.1%) of all OTUs (Figure S5). The ratio between these two classes of *Proteobacteria* appeared to be affected by the presence of oil and/or oil + Corexit. In the mesocosms amended with hydrocarbons (i.e., WAF, CEWAF, and DCEWAF), *Gammaprotebacteria* were generally between two and six-fold more abundant than *Alphaproteobacteria*. In contrast, the opposite was observed in the Control mesocosms; the abundance of *Alphaproteobacteria* was typically double that of the *Gammaproteobacteria*. This difference was driven largely by increases in relative abundance of the order *Alteromonadales* and a decrease in the relative abundance of the family *Rhodobacterales* in the treatments amended with hydrocarbons compared to the control (Figure S5). After the *Proteobacteria*, only *Bacteriodetes* and *Thaumarcheota* were present in significant numbers. *Bacteriodetes* initially comprised 2%-5% of the total community in each mesocosm. Over the following 72 h, their relative abundance steadily increased to 8–11% in the mesocosms without Corexit (i.e., Control and WAF), but remained static in the treatments containing the dispersant. Similarly, *Thaumarchaeota* were also present in all mesocosms, though their abundance appeared to be unaffected by either oil or Corexit®: A single OTU (OTU19; *Nitrosopumilus*) was present in all samples between 1 and 3% relative abundance and did not vary significantly between treatments.

All four mesocosm treatments harbored numerous bacterial taxa known or suspected to degrade hydrocarbons. Observed bacterial families primarily included *Alteromonadaceae* (*Median* 19.2%), *Rhodobacteraceae* (16.0%), *Piscirickettsiaceae* (8.0%), *Alcanivoracaceae* (8.0%), *Oceanospirillaceae* (5.7%), *Cellvibrionaceae* (3.9%), *Pseudomonadaceae* (2.9%), *Hyphomonadaceae* (2.2%), and *Saprospiraceae* (0.9%) (Figure S6). Among these families, abundant genera (defined as having ≥1% relative abundance) included *Aestuariibacter, Methylophaga, Alcanivorax, Marinobacter, Alteromonas, Pseudomarvicurvus, Glaciecola, Cycloclasticus, Oleibacter, Ponticaulis, Phaeodactylibacter, Neptuniibacter*, and *Pseudospirillum*. Detailed information of the relative abundances of OTUs within each treatment and time point is tabulated in the supplemental material (Data Sheet 1).

**Figure 3:**
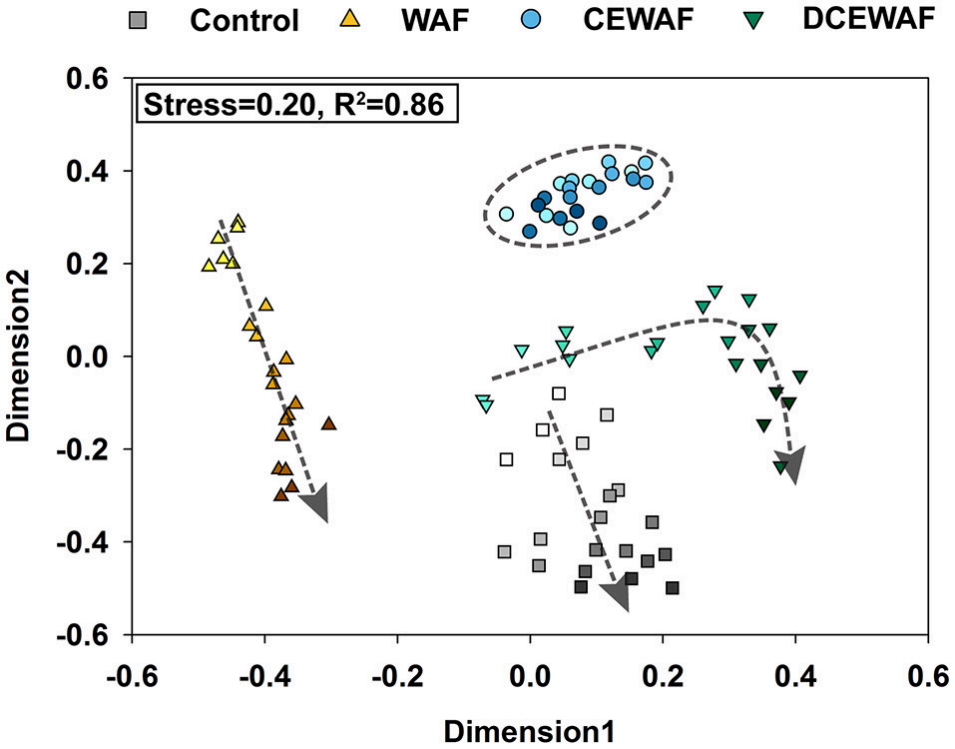
NMDS plot of the shifts in the microbial community structure observed in the four mesocosm treatments. Darker colors represent later time-points for each treatment, which is further highlighted with the overlaid arrows, which indicate the direction of community succession over time.

NMDS and analysis of molecular variance (AMOVA) indicated significant differences in community structure between the four mesocosm treatments [*F*_(3, 80)_ = 48.04, *p* < 0.001] (Figure 3). Given that all of the mesocosms were prepared from the same original seawater sample, we expected that the microbial community structure would be similar at first and then diverge over time as they responded to each treatment. However, the differences in community structure between the four treatments was already significant at the first time point (0 h) [*F*_(3, 8)_ = 18.94, *p* < 0.001], indicating the microbial community response to oil and/or Corexit® was quite rapid and likely began during the preparation of the WAF and CEWAF (previous ~24 h). Nonetheless, with the exception of the CEWAF treatment, community structure continued to shift over time within the mesocosms, representing continuing community succession throughout the experimental time-course (Figure 3).

**Figure 4:**
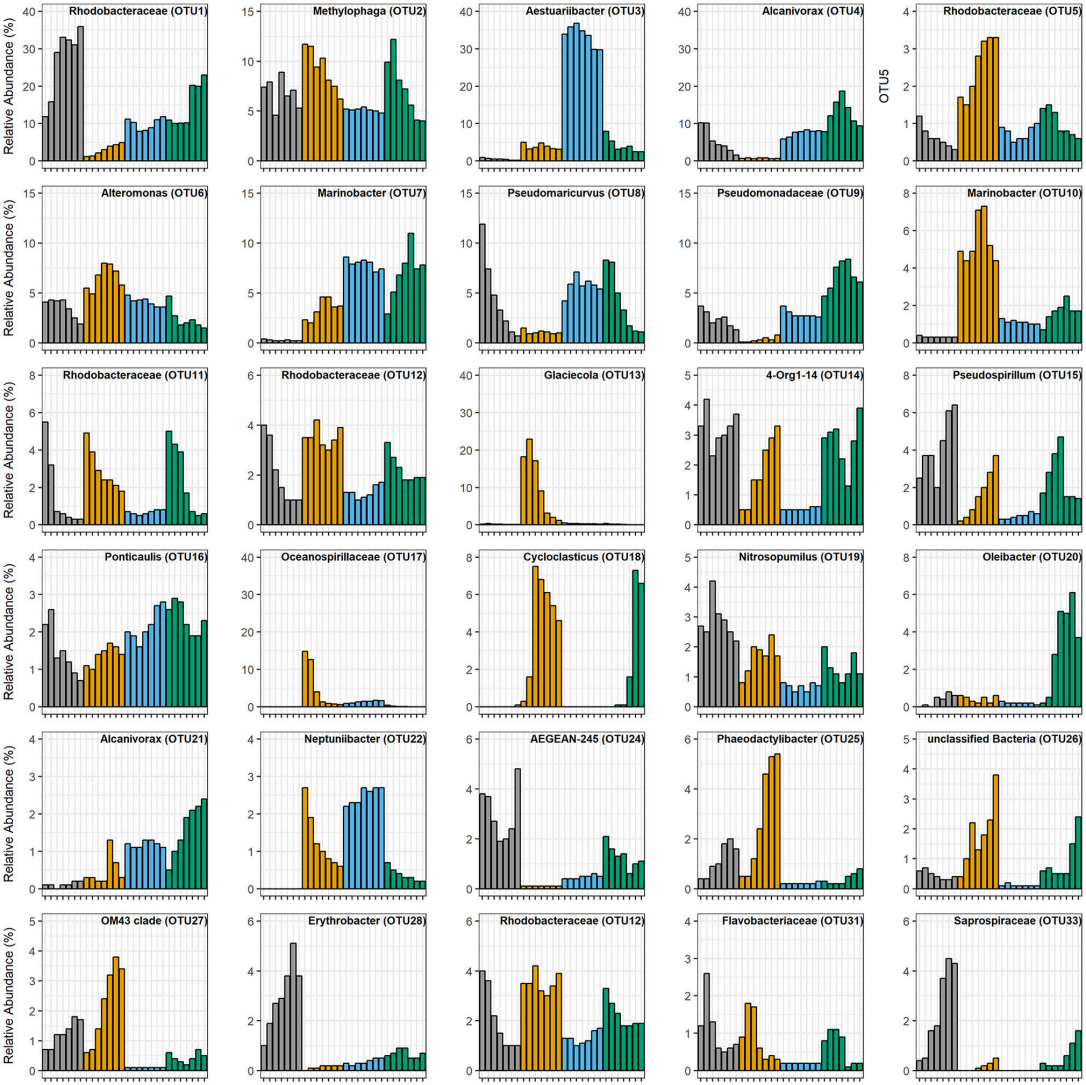
Relative abundances of the 30 most abundant OTUs. Each bar is the average of triplicate treatments. Tick marks on the x-axes are the same as those in Figure 1, demarking each experimental time point, taken every 12 h. Color key is the same as Figure 1: gray is Control, orange is WAF, blue is CEWAF, and green is DCEWAF. When no data is presented, this reflects the absence of the OTU in that treatment.

The community shift in the Control mesocosms (initial EOE = below detection) was largely driven by the growth of a single OTU (OTU1) representing an uncultured *Alphaproteobacteria* within the family *Rhodobacteraceae* (Figure 4). At 0 h this OTU comprised 11.8% ± 1.5% (*M* ± *SD*) of the total microbial community within the Control mesocosms, but quickly bloomed over the following 72 h to become the overwhelmingly dominant OTU (35.9% ± 5.9% of all sequences). This outgrowth of OTU1 was accompanied by the simultaneous relative decrease of two other *Rhodobacteraceae* OTUs (OTU11 and OTU12) as well as several initially abundant *Gammaproteobacteria* OTUs: *Pseudomaricurvus* (OTU8; −18×), *Alcanivorax* (OTU4; −6.4×), and *Alteromonas* (OTU6; −2.2×).

**Figure 5:**
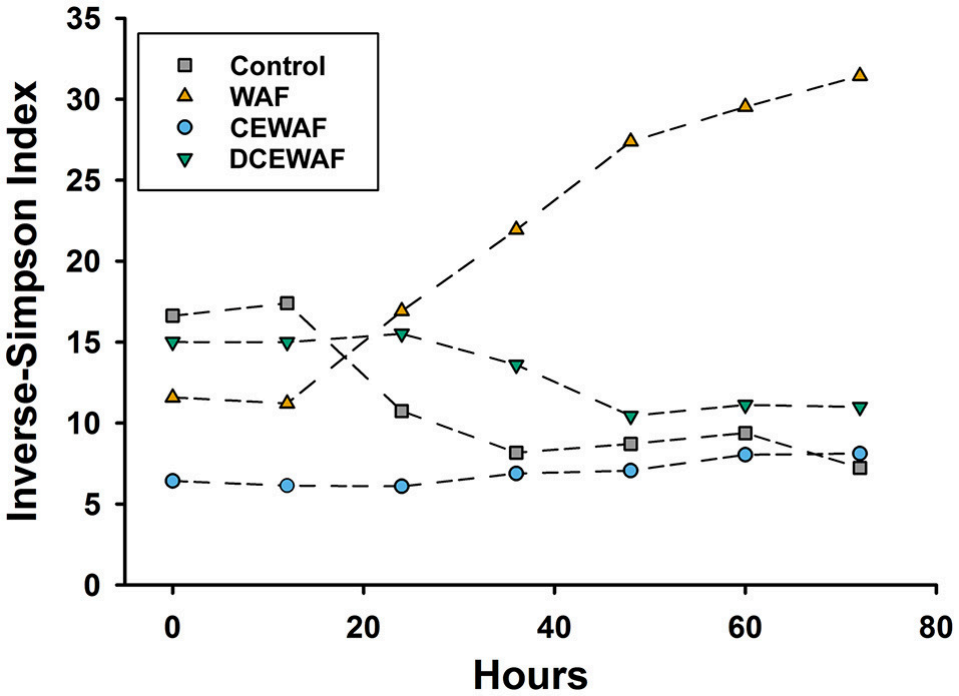
Observed changes in phylogenetic diversity, calculated as the inverse-Simpson index, over time for each of the mesocosm treatments.

The WAF mesocosms (initial EOE = 0.26 mg L^−1^) were the only ones wherein the microbial community diversity (calculated as inverse-Simpson index) increased over time (Figure 5). This increase was driven by the rapid decline of some of the initially abundant OTUs accompanied simultaneously by the growth of several initially rare taxa. For example, OTU13 (*Glaciecola*) and OTU17 (unclassified *Oceanospirillaceae*) were the most abundant OTUs (18.2% ± 1.7% and 14.8% ± 1.3%, respectively) during the first 24 h, but decreased to ≤ 1% relative abundance over the following 48 h. Simultaneously, several initially rare OTUs (≤0.5%) increased substantially in abundance: OTU85 (*Porticoccus*), 278-fold; OTU37 (*Alcanivorax*), 116-fold; OTU43 (*Pseudospirillum*), 20-fold; OTU18 (*Cycloclasticus*), 18-fold; OTU15 (*Pseudospirillum*), 15-fold; OTU68 (*Litoricola*), 15-fold; and OTU25 (*Phaeodactylibacter*), 12-fold (Figure 4, Figure S7).

In the CEWAF mesocosms (initial EOE = 41.53 mg L^−1^), the microbial community composition and structure remained essentially static between the first and last time point (*p* = 0.050; Figures 2–4). No major changes in relative abundance were observed for any abundant or rare OTU. Diversity was the lowest among the four treatments (Inverse-Simpson = 6.95 ± 0.80; Figure 5 and Table S1), with ~34% of the total community represented by a single OTU (OTU3) closely related to *Aestuariibacter aggregatus*. Other abundant phylotypes included *Pseudomaricurvus* (8.0% ± 0.5%), *Marinobacter* (6.1% ± 0.1%), *Alcanivorax* (8.6% ± 0.8%), and *Methylophaga* (4.2% ± 0.4%).

The DCEWAF mesocosms (initial EOE = 2.74 mg L^−1^) were similar to the Control mesocosms in that OTU1 (unclassified *Rhodobacteraceae*) was also highly abundant, but its pattern of growth was different. Instead of a continuous increase in abundance throughout the experiment, OTU1 remained stable at 10.3 ± 0.4% relative abundance for the first 48 h, and then quickly doubled in population sometime between 36 and 48 h (Figure 4 and Data Sheet 1). Furthermore, the DCEWAF communities uniquely saw several OTUs exhibit early growth followed quickly by a decrease in relative population (Figure 4). For example, OTU4 (*Alcanivorax*) initially comprised 7.8 ± 0.8% of the community at 0 h, then bloomed to 18.7 ± 2.4% over the following 36 h, but then decreased again to 9.4 ± 0.9% by the final time-point (72 h). Similarly, OTU20 (*Oleibacter*) abundance was initially quite rare (0.20% ± 0.02%), then multiplied over 30-fold to 6.1 ± 2.0%, and finally decreased to 3.7 ± 0.2% during the last 24 h. Similar to the WAF treatment, OTU18 (*Cycloclasticus*) was initially very rare (<0.02%) but then multiplied in abundance nearly 450-fold to 6.9 ± 2.9% within 72 h. Ultimately, the DCEWAF mesocosms exhibited a similar decrease in diversity to the Control mesocosms wherein OTU1 became the most abundant taxon (Figure 5). However, this decline was noticeably slower in the DCEWAF. This pattern of community succession is visible in the NMDS plot (Figure 3) wherein the DCEWAF communities clearly diverge away from those in the Control mesocosms for the first 36 h and then later begin to shift (downwards) back toward them as similar taxa are becoming abundant (i.e., OTU1).

**Figure 6:**
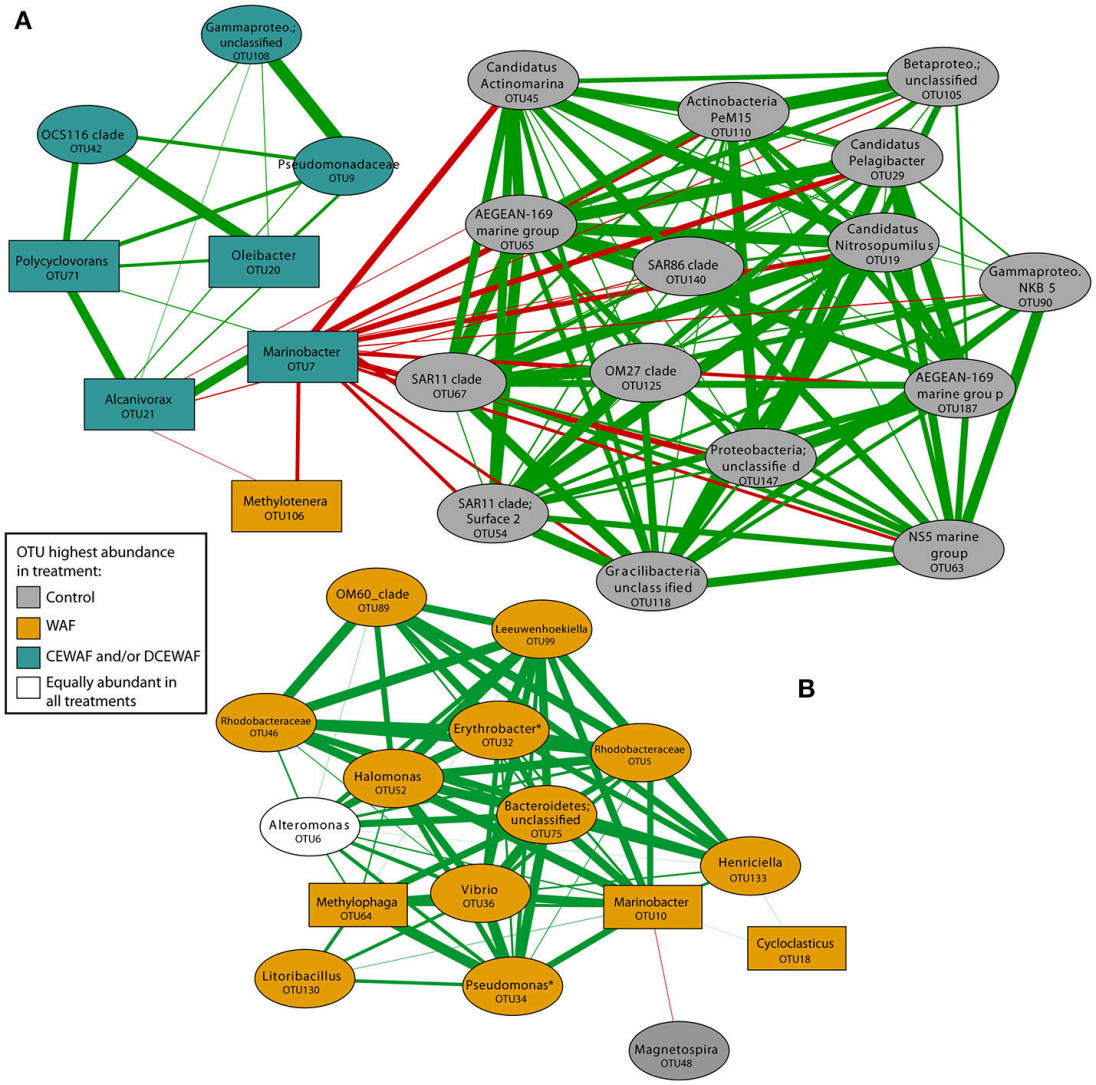
Correlation network analysis of OTUs within the mesocosm experiment. OTU nodes are colored according to the treatment in which they were most abundant. Rectangular nodes indicate a known or putative hydrocarbon degrading taxon. Asterisks denote taxa which contain both hydrocarbon degrading and non-hydrocarbon degrading members. The width of the lines connecting the nodes are proportional to correlation strength (from 0.70 to 0.99) with red and green lines indicating a negative or positive correlation, respectively. For simplicity of viewing, we only display OTUs that display one-step correlations to OTU7 and OTU20 **(A)** and OTU10 **(B)** to highlight connections between putative hydrocarbon degraders prevalent in WAF and Corexit amended treatments.

Correlation network analysis identified several community structural patterns between individual microbial OTUs within the mesocosms. For example, microbial taxa typically found in seawater (e.g., SAR11, SAR86, *Pelagibacter, Nitrosopumilus*) were strongly correlated with each other and most abundant in the Control treatment (Figure 6A). These taxa further exhibited strong negative correlations with several taxa of known hydrocarbon-degraders, including *Marinobacter* (OTU7), *Alcanivorax* (OTU21), and *Polycyclovorans* (OTU71), all of which were predominantly abundant in the CEWAF and DCEWAF treatments. In contrast, a separate OTU of *Marinobacter* (OTU10) was instead most abundant in the WAF treatment and had a strong positive correlation with several other taxa (Figure 6B), many of which are known or putatively contain hydrocarbon degrading species (i.e., *Methylophaga, Pseudomonas, Halomonas, Erythrobacter*, and *Cycloclasticus*).

## Discussion

In marine surface waters, the interactions between phytoplankton, prokaryotic microbes, and their exudates play an instrumental role in determining the fate of oil after its release (Quigg et al., 2016). However, current understanding of these interactions and their mechanisms is poorly resolved. The overall goal of our project is to investigate the impacts of oil and dispersants on marine surface water microbial communities and elucidate how these impacts influence the formation of marine oil-snow (MOS). Through the use of laboratory mesocosms, we were able to observe changes in microbial community composition and structure with substantially greater detail, temporal resolution, and experimental control than possible with field measurements. To our knowledge, this is the first study to monitor in significant detail the early response (within the first hours and days) of marine surface water microbial communities to oil and/or dispersant.

**Figure 7:**
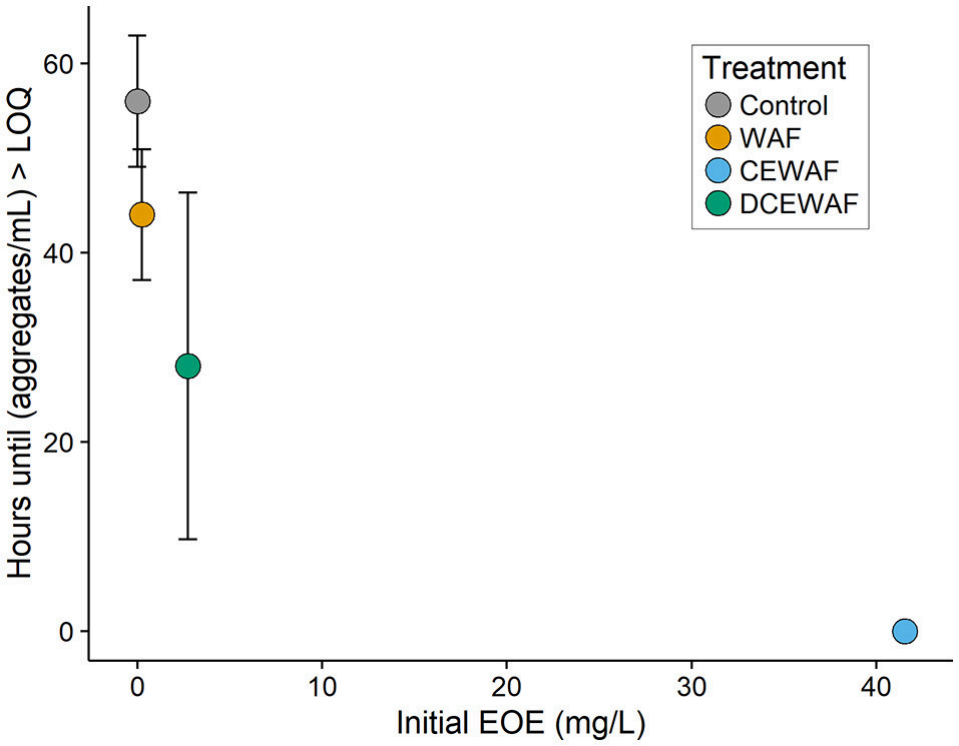
Time until a significant increase in aggregate abundance was observed within the mesocosms, plotted vs. the concentration of oil (EOE mg L^−1^). This was defined as the moment when the average aggregate concentration became greater than the limit of quantification (calculated here as *M* + 3·*SD*). Error bars represent standard deviation.

When exposed to oil, many hydrocarbon-degrading taxa will produce considerable amounts of EPS in an effort to emulsify the oil and increase its bioavailability (Head et al., 2006). Consistent with this notion, we observed that the first appearance of an increased aggregate abundance was directly related to the amount of oil in the mesocosms (Figure 7). WAF and CEWAF are heterogeneous mixtures of both dissolved hydrocarbons and microscopic oil droplets and as such contain more droplets at higher concentrations (Singer et al., 2000). These micro-droplets present a substrate surface for attack and colonization by hydrocarbon-degrading microorganisms, thus potentially triggering EPS production and subsequent aggregate formation. Indeed, we observed the highest abundances of micro-aggregates in mesocosms containing Corexit (Figure 1, middle), whose emulsifying properties would have dramatically increased the amount of suspended oil droplets within those treatments. Supporting this hypothesis, we found that many of the micro-aggregates observed in this study visibly contained droplets of oil at their center (Figure S2, Video S1). These micro-aggregates (10–200 μm) are generally smaller than MOS, which has been operational defined as being >0.5 mm. It is unknown if these micro-aggregates continue to increase in size and/or join together with time, or if they remain microscopic and potentially function as niches for hydrocarbon-degrading microorganisms. Our observations are consistent with those of Baelum et al. (2012), who noticed microbial cells began to aggregate within 2 days upon addition of oil to water. Furthermore, our findings are also similar to those of Kleindienst et al. (2015b), who found macroscopic MOS flocs were most abundant, largest, and formed the most rapidly in microcosms containing Corexit. Other mesocosm experiments performed by our own research consortia have detected microbe-cell aggregates (Quigg et al., 2016), but this was the first effort to quantify their relationship to concentration of accommodated oil.

Many of the microbial taxa we observed are similar to those seen during other recent microbiological surveys of environments affected by the DWH or Prestige oil spills, such as beaches (Jiménez et al., 2007; Alonso-Gutiérrez et al., 2009; Kostka et al., 2011) or deep seawater (Redmond and Valentine, 2012; Dubinsky et al., 2013). Similar to these studies, we also found the microbial communities within the mesocosms exhibited complex successional patterns involving both the population growth and decline of various hydrocarbon degraders. These compositional changes likely reflect the community transitioning through a complex series of various oil substrates and/or phytoplankton exudates. In our study, some of these changes occurred in just a few hours after experiment initiation, indicating that microbial communities in warm marine surface waters respond rapidly to oil and/or dispersant exposure.

One explanation for these rapid community shifts is that hydrocarbon-degrading bacteria were a significant fraction of the indigenous microbial community. Indeed, some of the bacterial taxa (e.g., *Alcanivorax, Alteromonas, Methylophaga, Rhodobacteraceae*) we initially observed within the Control mesocosms are known or suspected to contain hydrocarbon-degrading members. Multiple reports over the last 35 years have demonstrated that environments which have been chronically exposed to petroleum products tend to harbor microbial communities adjusted to the presence of hydrocarbons and contain correspondingly higher concentrations of hydrocarbon-degrading species (Griffiths et al., 1981; Carman et al., 1996; Geiselbrecht et al., 1998; Coulon et al., 2007; Hazen et al., 2010; Das and Chandran, 2011). In the Gulf of Mexico, an estimated 2.53–9.48 × 10^4^ m^3^ of oil (≈10% of that released during the DWH spill) is discharged each year by natural oil seeps (MacDonald et al., 2015) and the Galveston Bay area has historically been exposed to petroleum pollution from decades of intensive industrial and shipping activity (Santschi et al., 2001) as well as a recent oil spill event (Williams et al., 2017). In this context, the rapid microbial responses we observed may be reflective of what occurs in marine environments frequently exposed to hydrocarbons.

Many of the abundant or blooming OTUs we observed in the oil-amended mesocosms e.g., *Marinobacter, Methylophaga, Cycloclasticus, Alcanivorax, Rhodobacteraceae, Litoricola* belong to taxa which contain known or putative hydrocarbon degrading members and were detected *in situ* during the DWH oil spill or other oil-related microbiology studies (Hazen et al., 2010; Vila et al., 2010; Gutierrez et al., 2011, 2013b; Kostka et al., 2011; Lai et al., 2011; Dubinsky et al., 2013; Engel and Gupta, 2014; Yang et al., 2016). Many have also been detected in other oil-rich or polluted environments (Table S2). Notably absent however from our study were species of *Colwellia*. This genus of psychrophilic bacteria was consistently observed in great abundance during the DWH oil spill (Gutierrez et al., 2011; Redmond and Valentine, 2012; Dubinsky et al., 2013) and is believed to have played a major role in the degradation of oil within colder, higher pressure (11 MPa) subsurface waters. However, the near complete absence of this genus within our mesocosms (<0.001% total relative abundance) is not unexpected considering they were prepared with warmer (~25°C) surface waters at atmospheric pressure. This observation is consistent with Redmond and Valentine's (2012) evidence that temperature plays a major role in structuring the composition of a microbial community responding to hydrocarbons.

We also observed other potential hydrocarbon-degrading taxa which have not been detected in abundance in any of the previous DWH studies. For example, OTU85 is 100% identical to *Porticoccus hydrocarbonoclasticus* (Table S2), a hydrocarbon degrader isolated from the marine dinoflagellate *Lingulodinium polyedrum* and identified on cultures of other dinoflagellates and diatoms (Gutierrez et al., 2012). There is evidence that some phytoplankton (i.e., dinoflagellates, diatoms, and coccolithophores) adsorb PAH molecules from the environment onto their cell surfaces (Kowalewska, 1999; Binark et al., 2000) and many phytoplankton species synthesize hydrocarbons directly (Chisti, 2007; Schirmer et al., 2010), creating a niche for hydrocarbon-degrading bacterial epibionts (Allers et al., 2007; Gutierrez et al., 2011, 2012, 2013a). The blooming of a phytoplankton-associated, hydrocarbon degrading bacterium in our experiments supports this hypothesis and highlights the importance of bacteria-phytoplankton interactions in the microbial response to oil spills.

A few bacterial taxa not known to metabolize hydrocarbons also appeared to thrive in the WAF mesocosms [i.e., *Pseudospirillum* (OTU43), *Phaeodactylibacter* (OTU25), OM43 clade (OTU27), and subsection III cyanobacteria (OTU78)]. It is possible that these taxa were simply unaffected by the presence of oil or were associated with phytoplankton within the mesocosms. This latter possibility is especially likely for *Phaeodactylibacter*, considering the type species for this genus has been isolated from marine algae (Chen et al., 2014; Lei et al., 2015). Likewise, OM43 is a clade of obligate methylotrophic marine bacteria commonly associated with phytoplankton blooms (Morris et al., 2006; Giovannoni et al., 2008; Sowell et al., 2011; Huggett et al., 2012). Nevertheless, it is also possible that some of these taxa, although not previously described, were indeed involved in the degradation of oil within the WAF mesocosms. Given the enormous range of different hydrocarbon substrates found within oil, the ability to degrade hydrocarbons is a fairly unspecific and thus somewhat unexceptional phenotype among microbial lineages. As such, the role of these taxa in hydrocarbon degradation should not be entirely rejected based on phylogeny alone.

Most of the abundant (defined here as ≥1% total relative abundance) taxa observed in the CEWAF mesocosms are also well-known or suspected hydrocarbon degraders. For example, *Alcanivorax* spp. and *Marinobacter* spp. are thought to use saturated alkanes exclusively as their carbon source (Gauthier et al., 1992; Yakimov et al., 1998, 2007; Head et al., 2006) while *Alteromonas* spp. and *Neptuniibacter* spp. are believed to predominately degrade aromatic-hydrocarbons (Arahal et al., 2007; Dombrowski et al., 2016). The closest known relative of the most abundant taxon from the CEWAF mesocosms (100% similarity to *Aestuariibacter* sp. Table S2) has been demonstrated to degrade crude oil (Wang et al., 2014), but its specificity, if any, for either alkane or aromatic hydrocarbons is unknown. Additionally, some of these taxa were possibly enriched because the abundant quantity of oil droplets in the CEWAF treatment provided a readily available substratum for colonization. Indeed, bacteria affiliated with the *Rhodobacteraceae* and *Alteromonadaceae*—of which many OTUs in this treatment belong—are pioneer surface colonizers of particles and other submerged surfaces in marine waters (Dang and Lovell, 2000, 2016).

The lack of an observed change in the composition or structure of the microbial community within the CEWAF mesocosms was likely a factor of both hydrocarbon concentration and time. Due to the use of Corexit, a significantly larger fraction of oil was accommodated into the seawater and the initial concentration of oil within these mesocosms was more than two orders of magnitude higher than the WAF mesocosms (Figure 2). These concentrations (~42 mg L^−1^) are likely near the extreme upper limit of those which occur *in situ* immediately after dispersant application, before dispersed oil rapidly dilutes into the water column. Thus, one possible explanation for the static nature of the microbial community structure within the CEWAF mesocosms may be that alkanes and other components of oil which are typically degraded first by microbial consortia were persisting at saturating concentrations over the entire experimental time-course. Hence, the dynamic community succession of various aliphatic- and aromatic-hydrocarbon degrading taxa typically observed during marine oil spills (Kasai et al., 2002a,b; Röling et al., 2002, 2004; Head et al., 2006; Teira et al., 2007) was delayed beyond our relatively short experimental timeframe (72 h). This hypothesized persistence of alkanes is supported by the qPCR results, in which the abundance of cytochrome P450, a gene related to aerobic alkane degradation, did not diminish over time in the Corexit-amended treatments whereas it did in the others (Figure 1). Further lending support to this hypothesis is our observation that *Cycloclasticus*-related OTUs were nearly completely absent within the CEWAF mesocosms, but were enriched in the WAF and DCEWAF mesocosms. In particular, OTU18 was enriched in both WAF and DCEWAF after 36 and 60 h, respectively, but not in CEWAF (Figure 4), suggesting a succession toward PAH degradation in WAF and DCEWAF toward the latter part of our experiment. *Cycloclasticus* spp. are obligate PAH degraders (Dyksterhouse et al., 1995) commonly found in oil-contaminated marine environments (Kasai et al., 2002a), including deep-sea waters in the Gulf of Mexico after the DWH spill (Valentine et al., 2010; Mason et al., 2012; Redmond and Valentine, 2012; Kleindienst et al., 2016a). However, due to their specificity for the more recalcitrant PAHs, they struggle to compete with alkane degraders for nutrients and typically do not reach large populations sizes until after alkane-hydrocarbon concentrations have become more limiting (Kasai et al., 2002a,b; Röling et al., 2002).

Beyond community membership, we also noted some key similarities and differences in community structure between our study and previous work regarding the impact of Corexit on certain bacterial taxa. For example, while we did observe relatively abundant (~3–5% relative abundance) *Marinobacter*-related OTUs in our WAF treatments, none were found to be the dominant microbial taxa at any time, nor did we find their abundance to decline in the presence of dispersants. In fact, of the two abundant *Marinobacter*-related OTUs we observed, OTU7 and OTU10, one displayed a higher relative abundance in the Corexit-amended treatments (CEWAF and DCEWAF, Figures 3, 5) while the other was more abundant in the WAF treatments. This contrasts with the results of Hamdan and Fulmer (2011) and Kleindienst et al. (2015b), who both reported *Marinobacter* species are negatively impacted by dispersants. Instead, our data is consistent with the recent findings of Techtmann et al. (2017) who observed both stimulatory and inhibitory, temperature dependent effects of Corexit for different OTUs of *Marinobacter*. We also observed similar contrasting effects on OTUs of *Alcanivorax* and *Rhodobacteraceae*. *Alcanivorax* OTUs 4 and 21 generally displayed higher relative abundances in dispersant-amended treatments (Figure 4) while OTU37 instead appeared sensitive to dispersed oil (Figure S7). Likewise, many of the *Rhodobacteraceae* OTUs displayed concentration-dependent responses to the Corexit-amended treatments: OTU46 was found almost exclusively in the WAF, OTU5 was most abundant in WAF with lower but generally equal relative abundances in CEWAF and DCEWAF, while OTU11 had similar relative abundance in the WAF and DCEWAF, but was notably depleted in the CEWAF (Figure 4, Figure S7). Similarly, *Cycloclasticus* (OTU18) only appeared sensitive to chemically-dispersed oil at high concentrations (i.e., CEWAF). Taken together, these observations are consistent with the hypothesis that the impact of Corexit on microorganisms is species-specific and concentration dependent.

A limitation of our experimental design, as well as previous studies which also used natural microbial assemblages, is that our data does not allow us to conclusively identify how Corexit impacts any specific microbial taxa. Some species may be directly sensitive to the dispersant itself (e.g., Hamdan and Fulmer, 2011) or are instead stimulated and utilize the dispersant as a substrate (e.g., Campo et al., 2013). Alternatively, other species could be unreactive to the dispersant but are predominantly impacted by the substantially higher oil concentrations which dispersant usage enable, either directly due to toxicity from the oil itself, or indirectly through increased competition for nutrients. Such may be the case in our study; using Corexit increased the amount of oil accommodated into the water column nearly 150-fold. Prince et al. (2017) showed that oil concentration is the principal factor explaining discrepancies in rates of oil biodegradation between different experiments. Our data support this hypothesis.

These confounding parameters as well as subtle differences in methodology between studies (e.g., direct oil amendment vs. WAF/CEWAF preparation, oil concentration, type of oil, etc.) likely explain why the impacts of oil and dispersants on microorganisms remain equivocal (Kleindienst et al., 2016b; Prince et al., 2016 comments). Lee et al. (2013) pointed out that most laboratory experiments utilize oil concentrations that are unrealistically high and do not account for the enormous dilution that occurs in the ocean. The initial oil concentrations within our WAF (~0.3 ppm) and DCEWAF (~3 ppm) mesocosms are consistent with the realistic conditions (<10 ppm after 24 h) proposed by Lee et al. (2013) and are among the lowest, by approximately an order of magnitude, when compared to most previous works (see Table S1 of Prince et al. (2017) for an excellent summary). They are also consistent with measurements from within the subsurface plume during the DWH event, where higher end EOE concentrations were 10 ppm and most samples above detection were 0.1–10 ppm (Wade et al., 2016). These lower oil concentrations in our experiment may explain some of the stark microbiological differences we observed in our experiment compared to previous studies. For example, within our WAF mesocosms, the outgrowth of several initially rare putative hydrocarbon degraders resulted in increased taxonomic diversity. In contrast, (Baelum et al., 2012) observed that oil input led to decreased diversity in their enrichments, but had an initial oil concentration of ~30 ppm in their oil-alone treatment. This high concentration is more comparable to our CEWAF mesocosms (~42 ppm) wherein we also observed decreased taxonomic diversity.

In summary, we found the microbial community response to oil (as WAF) and Corexit (as CEWAF and DCEWAF) in warm surface waters to be rapid, with significant taxonomic shifts occurring on the order of a few hours. With such community changes occurring on such a short time scale, this demonstrates that studies—particularly those which focus on warmer surface waters—may miss a significant portion of the early microbial community dynamics which occur shortly after an oil spill. This is probably especially the case in environments such as the Gulf of Mexico, where natural oil seepage has pre-adapted the resident microbial communities, at least in part, to the presence of hydrocarbons. We found evidence suggesting application of dispersants can lead to saturating concentrations of hydrocarbon substrates for oil-degrading microorganisms, which slows down community succession compared to non-dispersant amended treatments. However, it is important to point out that it is possible this effect may only occur at unrealistically high concentrations of dispersed oil or elevated dispersant to oil ratios (> 1:20) as we did not observe a similar impact on community succession in the DCEWAF treatments wherein the total oil and Corexit concentrations were more comparable to *in situ* conditions during the DWH spill. We also provide evidence that the application of Corexit catalyzes the formation of microbial aggregates by increasing the amount of oil droplets suspended in the water column. These microscopic microbial-oil aggregates are potentially the precursors to the abundant macroscopic MOS observed after the DWH spill, but this linkage remains to be proven. Hence, further study to elucidate why dispersants have seemingly confounding effects on marine snow formation at different size scales is necessary.

## Author contributions

SD, TW, AK, PS, AQ, and JS: Conceived and designed the experiments; SD, EW, VD, TW, AK, PS, AQ, and JS: Performed the experiments; SD, EW, and JS: Analyzed the data; SD, EW, VD, TW, AK, PS, AQ, and JS: Wrote the paper.

### Conflict of interest statement

The authors declare that the research was conducted in the absence of any commercial or financial relationships that could be construed as a potential conflict of interest.

## Supplementary material

Figure S1 Four treatments were prepared for the mesocosm experiment. From left to right: (1) Control, (2) WAF, (3) DCEWAF, (4) CEWAF.

Figure S2 Deconvoluted, z-stacked fluorescent micrograph of micro-aggregate collected from a CEWAF mesocosm water sample after 72 h. Cells were stained with DAPI and appear as bright blue. Autofluorescent oil-Corexit droplets are false-colored orange. Deconvolution was performed using the Zeiss Apotome structured illumination system on a Zeiss Axio Imager M2 microscope.

Figure S3 Rarefaction analysis of 16S rRNA libraries from the four mesocosm treatments. Colors become darker for later time points.

Figure S4 β-galactosidase activity (nM/day) over time for each of the mesocosm treatments. Columns represent the pooled mean of triplicate measurements with error bars representing the pooled standard error.

Figure S5 Relative abundances of the various microbial lineages observed in the four mesocosm treatments. Each bar is the average of triplicate treatments. Taxa with relative abundances <1% were combined into the minor members category.

Figure S6 Relative abundances of bacterial families with known or putative hydrocarbon-degrading members observed in the four mesocosm treatments. Each bar is the average of triplicate treatments.

Figure S7 Relative abundances of 16 OTUs which displayed large increases or decreases in relative abundance. Each bar is the average of triplicate treatments. Tick marks on the x-axes are the same as those in Figure 4, demarking each experimental time point, taken every 12 h. Color key: gray is Control, orange is WAF, blue is CEWAF and green is DCEWAF.

Table S1 Comparison of alpha diversity metrics observed in four different mesocosm treatments using 16S rRNA hyper-variable V4 region sequence reads clustered with a 3% dissimilarity cutoff.

Table S2 Classifications of OTUs which exhibited large relative abundances or large changes in relative abundance over time.

Data Sheet 1 Number of reads and corresponding relative abundances of the OTUs within each treatment, at each time point.

Video S1 Three-dimensional rotation of a deconvoluted, z-stacked fluorescent micrograph of a micro-aggregate collected from a CEWAF mesocosm water sample after 72 h. Cells were stained with DAPI and appear as bright blue. Autofluorescent oil-Corexit droplets are false-colored orange. Deconvolution was performed using the Zeiss Apotome structured illumination system on a Zeiss Axio Imager M2 microscope.
