# Supplementary figures and images for: Rapid Formation of Microbe-Oil Aggregates and Changes in Community Composition in Coastal Surface Water Following Exposure to Oil and the Dispersant Corexit
Source: Front Microbiol. 2018 Apr 11;9:689. doi: 10.3389/fmicb.2018.00689 (PMC5904270; doi:10.3389/fmicb.2018.00689)

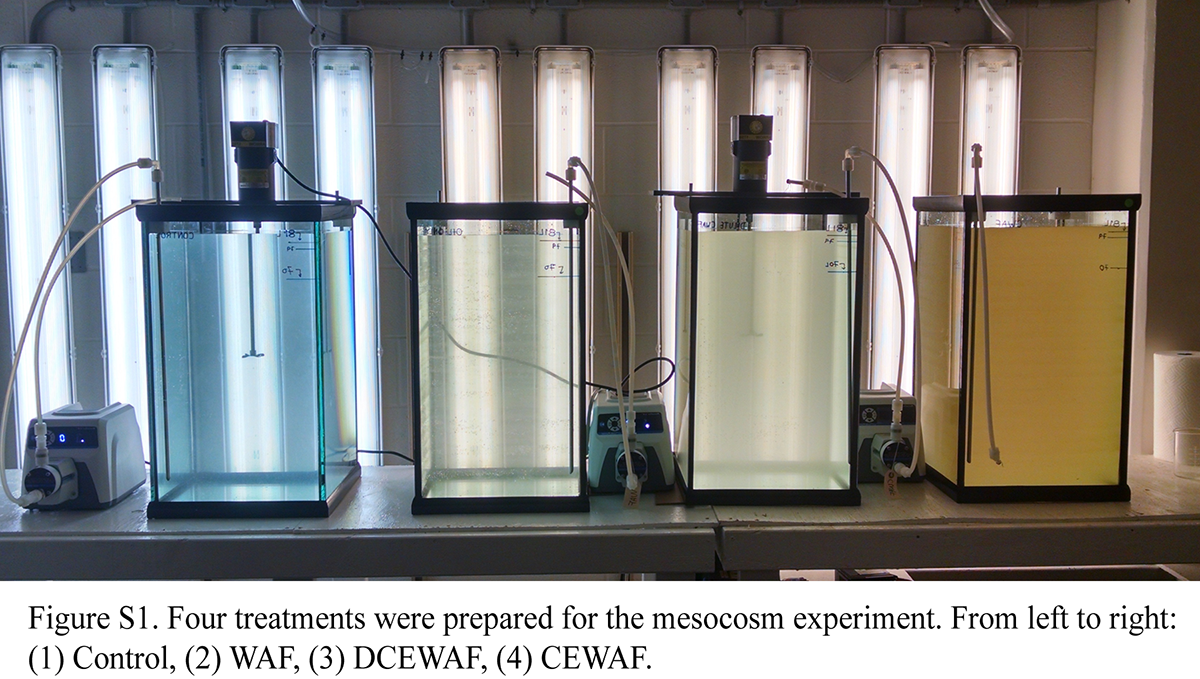

Supplement: Figure S1 — Four treatments were prepared for the mesocosm experiment. From left to right: (1) Control, (2) WAF, (3) DCEWAF, (4) CEWAF. [file Image1.TIF]

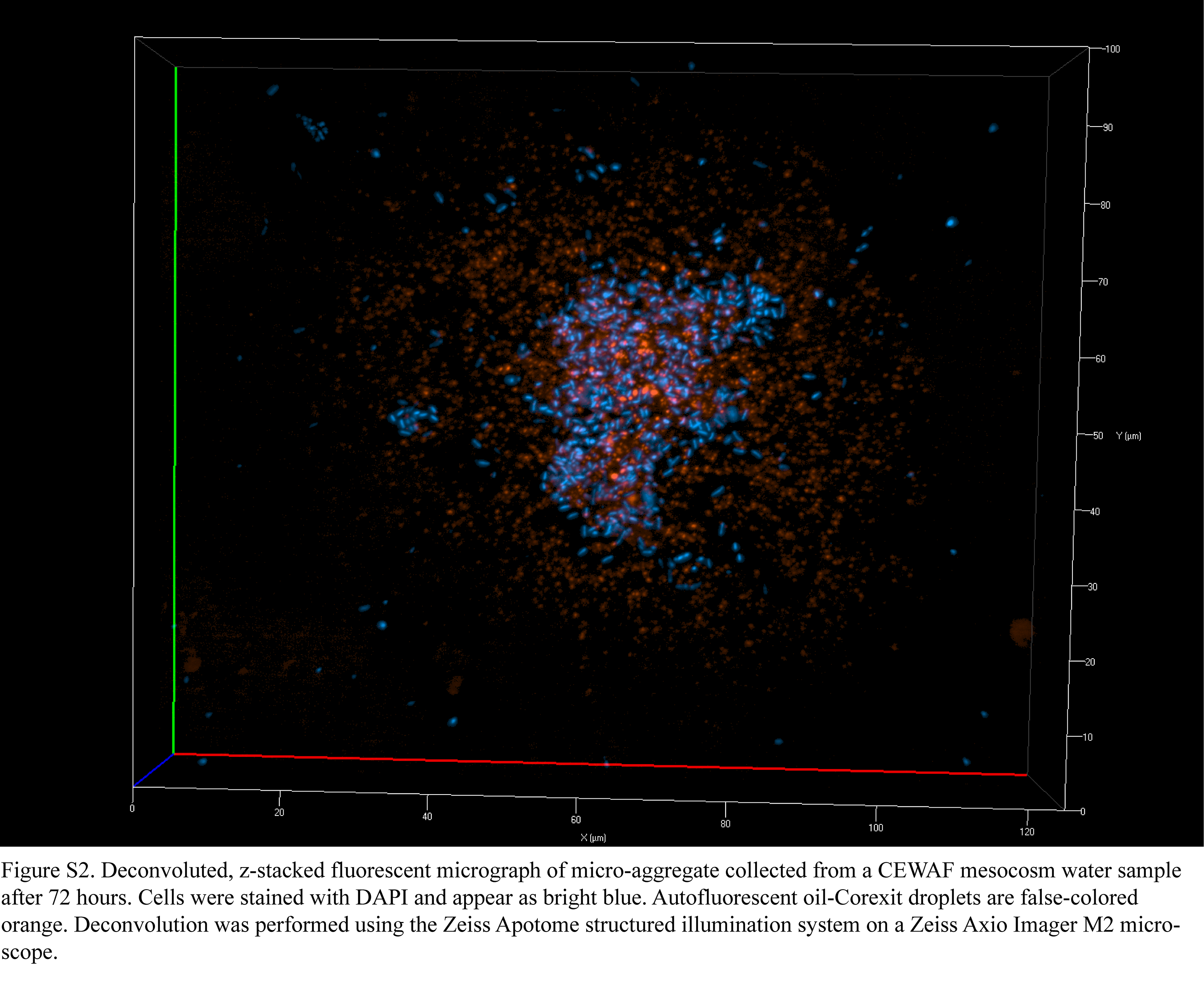

Supplement: Figure S2 — Deconvoluted, z-stacked fluorescent micrograph of micro-aggregate collected from a CEWAF mesocosm water sample after 72 h. Cells were stained with DAPI and appear as bright blue. Autofluorescent oil-Corexit droplets are false-colored orange. Deconvolution was performed using the Zeiss Apotome structured illumination system on a Zeiss Axio Imager M2 microscope. [file Image2.TIF]

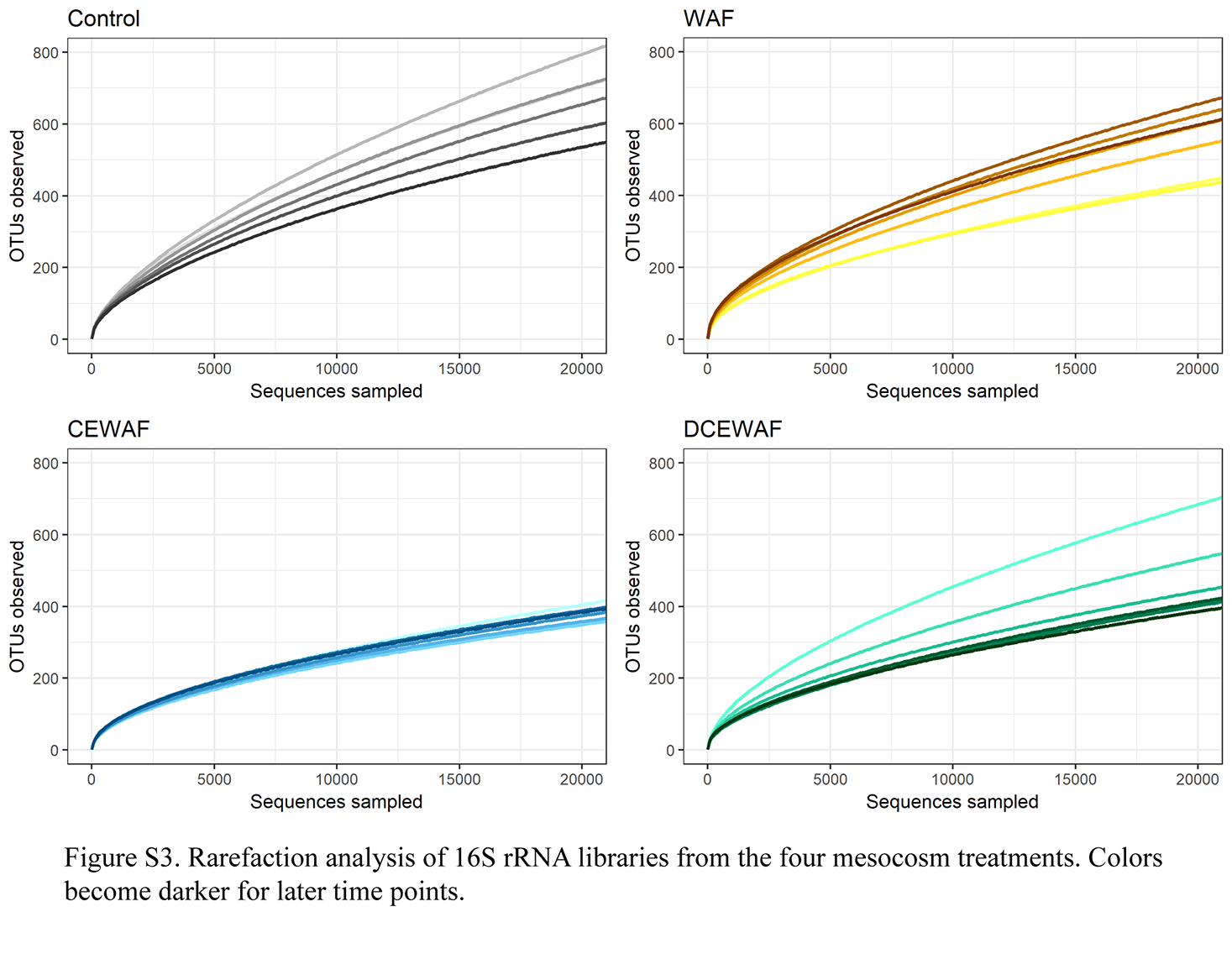

Supplement: Figure S3 — Rarefaction analysis of 16S rRNA libraries from the four mesocosm treatments. Colors become darker for later time points. [file Image3.TIF]

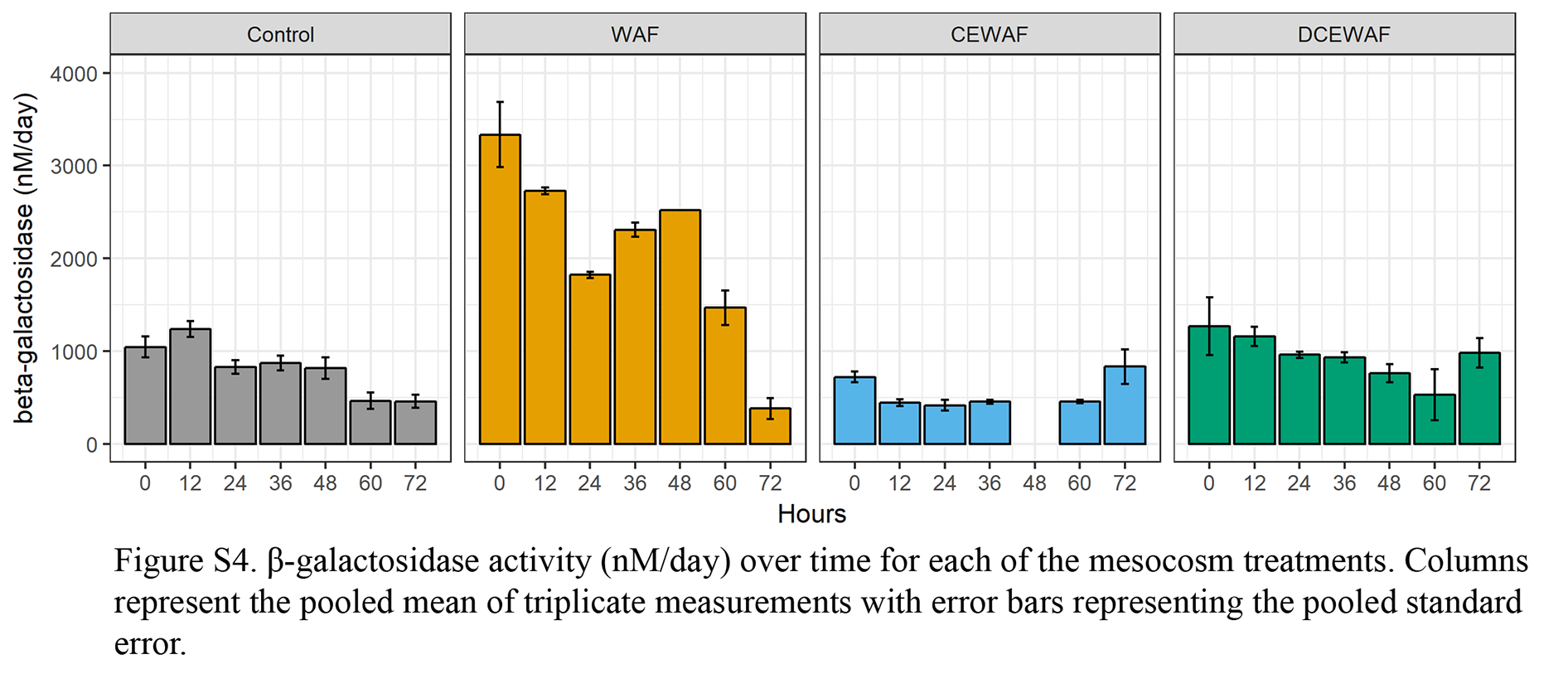

Supplement: Figure S4 — β-galactosidase activity (nM/day) over time for each of the mesocosm treatments. Columns represent the pooled mean of triplicate measurements with error bars representing the pooled standard error. [file Image4.TIF]

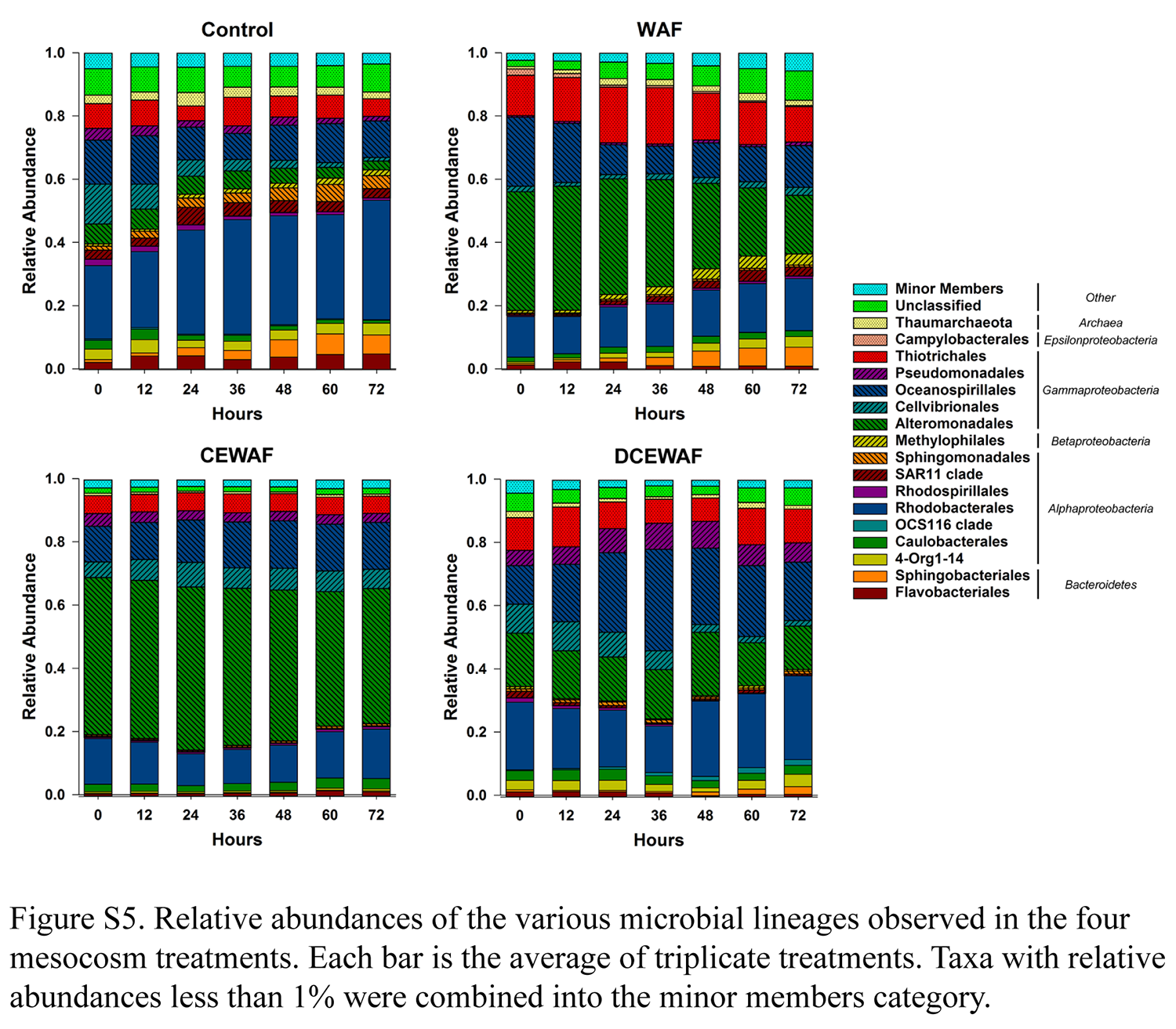

Supplement: Figure S5 — Relative abundances of the various microbial lineages observed in the four mesocosm treatments. Each bar is the average of triplicate treatments. Taxa with relative abundances <1% were combined into the minor members category. [file Image5.TIF]

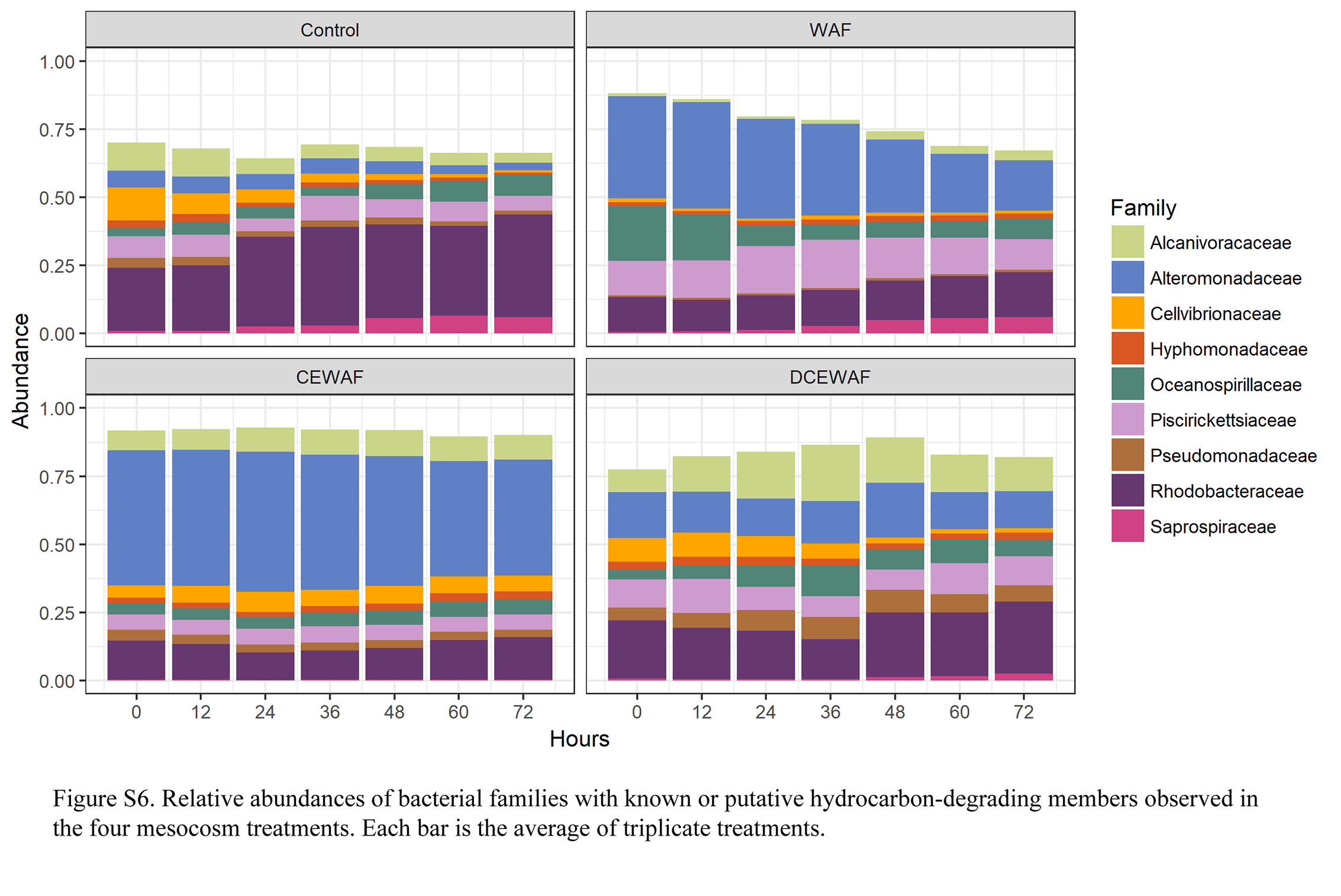

Supplement: Figure S6 — Relative abundances of bacterial families with known or putative hydrocarbon-degrading members observed in the four mesocosm treatments. Each bar is the average of triplicate treatments. [file Image6.TIF]

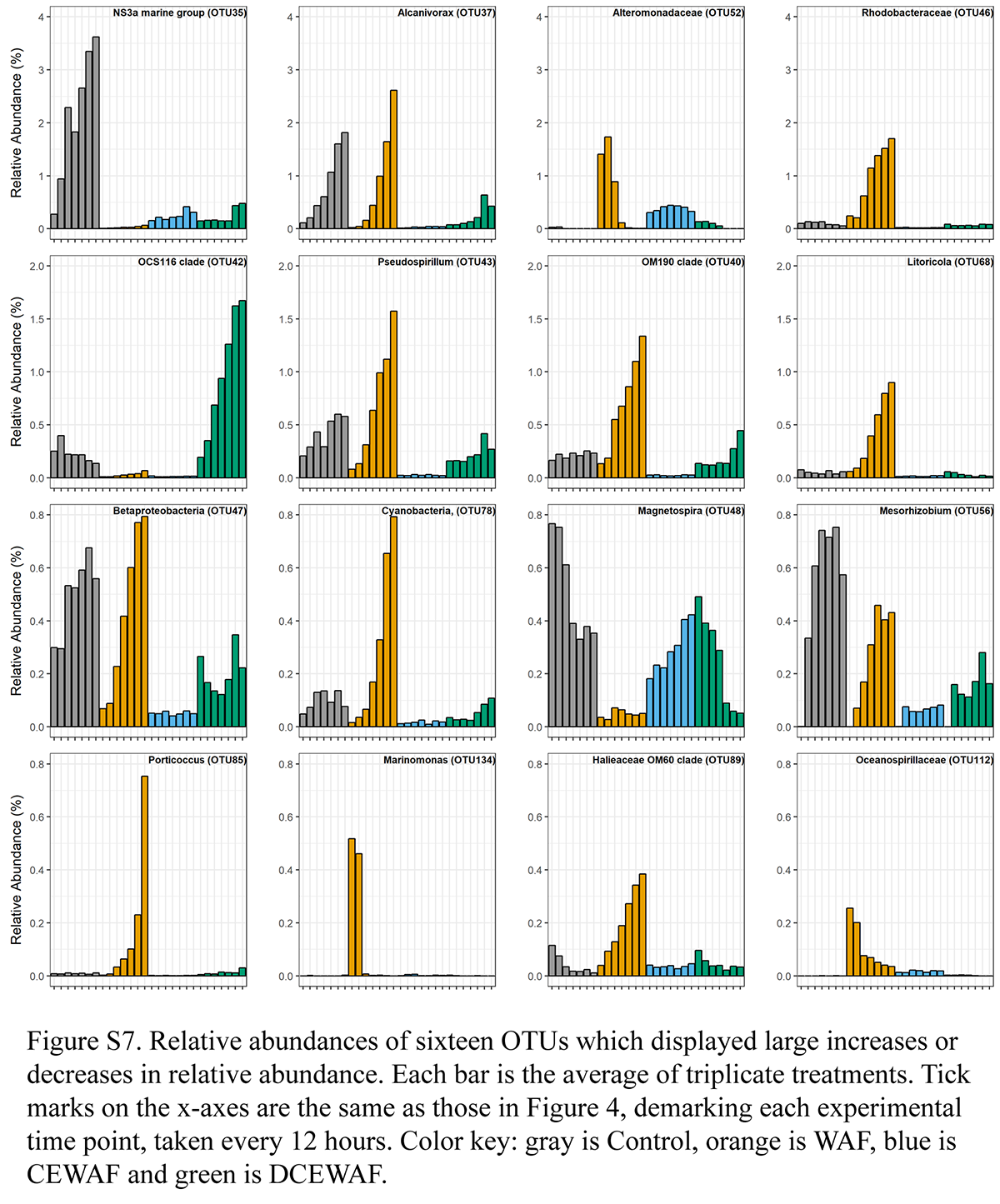

Supplement: Figure S7 — Relative abundances of 16 OTUs which displayed large increases or decreases in relative abundance. Each bar is the average of triplicate treatments. Tick marks on the x-axes are the same as those in Figure 4, demarking each experimental time point, taken every 12 h. Color key: gray is Control, orange is WAF, blue is CEWAF and green is DCEWAF. [file Image7.TIF]
